# Carob Tree: A Review of Traditional Uses, Medicinal Properties, and Future Perspectives in Sustainable Forestry

**DOI:** 10.3390/life16030448

**Published:** 2026-03-10

**Authors:** Abdelkader Gadoum, Abdelkader Difallah, Ahmed Adda, Othmane Merah

**Affiliations:** 1 Laboratory of Agronomy and Environment, Department of Agronomic Sciences, Forestry and Environment, Institute of Nature and Life Sciences, Tissemsilt University, Tissemsilt 38000, Algeria; 2 Laboratory of Biology and Organism Physiology, Faculty of Biological Sciences, University of Science and Technology Houari Boumediene, Algiers 16111, Algeria; 3 Laboratory of Agro-Biotechnology and Nutrition in Semi-Arid Zones, Department of Nutrition and Agri-Food Technology, Faculty of Nature and Life Sciences, Tiaret University, Tiaret 14000, Algeria; 4 Laboratory of Plant Biotechnology and Genetic Improvement, Department of Nutrition and Agri-Food Technology, Faculty of Nature and Life Sciences, Tiaret University, Tiaret 14000, Algeria; 5 Agro-Industrial Chemistry Laboratory (LCA), French National Research Institute for Agriculture, Food and Environment, Institut National Polytechnique de Toulouse, University of Toulouse, 31030 Toulouse, France; 6 Department of Biological Engineering, University Institute of Technology (IUT), University of Toulouse, 32000 Auch, France

**Keywords:** *Ceratonia siliqua* L., bioactive compound, gallic acid, flavonoids, antioxidant properties

## Abstract

The carob tree (*Ceratonia siliqua* L.) is indigenous to the Mediterranean basin, noted for its adaptability to biotic and abiotic stresses and its long history of use in traditional agroforestry systems. This review critically analyzes the phytochemical composition of carob, its traditional medicinal uses, and its contemporary applications in the cosmetic, pharmaceutical, and agri-food sectors. Particular attention is placed on the valorization of carob pods, seeds, and leaves, which are transformed into high-value products, including locust bean gum and polyphenol-rich extracts. Recent studies indicate that carob is a rich source of bioactive compounds, particularly phenolic acids and flavonoids such as gallic acid, chlorogenic acid, ellagic acid, catechins, quercetin, and luteolin. These compounds have primarily been investigated in vitro and in vivo, where they exhibited antioxidant, antimicrobial, and potential cardioprotective and gastrointestinal-related effects. This chemical diversity underscores their potential as a prime substitute for future nutraceutical and pharmaceutical applications. The review further addresses the ecological and socio-economic relevance of carob cultivation, particularly in countries such as Algeria, where reforestation and agro-industrial valorization remain underexploited despite their significant economic potential. Overall, this work highlights the need for a comprehensive and critical evaluation of carob-derived bioactive compounds and encourages further well-designed studies, especially clinical investigations, to better substantiate their health-related benefits while supporting sustainable use of this multipurpose species.

## 1. Introduction

The carob tree (*Ceratonia siliqua* L.) is native to the Mediterranean region, especially Algeria, where it contributes significantly to the maintenance of regional biodiversity [1]. Its distribution is largely concentrated in coastal and semi-arid environments, reflecting its remarkable adaptation to harsh climatic conditions [2]. The species thrives across a wide altitudinal range and is commonly found in semi-arid and arid areas, where it supports biodiversity conservation and soil stabilization [3].

The carob tree is increasingly considered a key species for reforestation and land restoration programs in regions affected by drought and soil degradation due to its exceptional tolerance to environmental stresses, which has earned it recognition as a symbol of resilience and an effective tool in the fight against desertification in Algeria [4,5]. Beyond its ecological importance, *C. siliqua* has been traditionally utilized across generations. Indigenous to the Near East, it has long been valued for its fruits, seeds, and therapeutic properties. Historically, carob pods provided an important source of nutrition for both humans and livestock [6,7].

In ancient civilizations, particularly in Egypt, carob pods were incorporated into pharmaceutical and dietary preparations and employed in the treatment of various gastrointestinal disorders [8]. In modern contexts, carob is widely utilized in the production of food products, including flour, syrups, and cocoa substitutes, due to its natural sweetness, high nutritional value, and rich dietary fiber content [9].

Ancient Greek and Roman societies acknowledged their therapeutic potential, particularly astringent tannins in the pods, which were commonly used to alleviate digestive discomfort [10]. In traditional medicine, carob has been employed to treat gastrointestinal disorders and as a demulcent for respiratory conditions [11]. These health benefits are typically derived from extracts or decoctions prepared from carob leaves and pods [12].

Nowadays, carob is attracting growing attention as a Mediterranean species of considerable ethnobotanical and pharmacological relevance [13,14]. Despite this rising interest, comprehensive reviews that integrate traditional knowledge with contemporary pharmacological findings remain scarce. Such integration is particularly important in the context of the bioeconomy, where carob represents both a promising alternative to cocoa and a valuable resource for pharmaceutical applications [15]. Recent studies have identified numerous bioactive compounds, including polyphenols and flavonoids, which have been linked—primarily through experimental studies—to anti-hyperglycemic, anti-inflammatory, and antioxidant activities [16,17,18].

The aim of this study is to compile and critically examine traditional carob-related practices worldwide, with a particular focus on the Mediterranean basin, where this species holds substantial socio-economic importance. By rigorously reviewing available scientific evidence on its health-related effects, this work seeks to establish clearer connections between ancestral uses and contemporary pharmacological research, while highlighting knowledge gaps and future research directions.

## 2. Methodology

A systematic review of the scientific literature was conducted, focusing on publications from 2017 and 2024. Studies published before 2017 were excluded, except for selected historical references that provided relevant quantitative data concerning the carob tree and its traditional or economic value.

The literature search was conducted using major academic databases, including PubMed, Scopus, and Web of Science, and additional sources were identified via publishers’ platforms, namely ScienceDirect and MDPI, to ensure comprehensive coverage of peer-reviewed publications.

A structured search strategy was developed to collect and analyze data relevant to the objectives of this review. Keywords included “*Ceratonia siliqua*”, “carob”, “nutritional value”, “antioxidant”, “anti-inflammatory”, “traditional use”, “sustainable agriculture”, “agroforestry”, “desertification”, and “cocoa substitute”.

To ensure rigor and relevance, predefined inclusion and exclusion criteria were systematically applied. Eligible studies included original research articles and review papers published in peer-reviewed journals, written in English or French, based on in vitro and/or in vivo experimental approaches; these studies had to address at least one of the following themes: (i) nutritional characteristics of carob, (ii) pharmacological or bioactive properties, or (iii) agricultural and environmental sustainability related to the carob tree.

The selection process was conducted in two stages. First, titles and abstracts of retrieved publications were screened according to the inclusion and exclusion criteria. Second, the full texts of selected articles were examined to confirm eligibility. Unpublished studies, conference proceedings, non-peer-reviewed publications, and studies focusing on species other than *Ceratonia siliqua* were excluded.

For analytical clarity, extracted data were organized into three main thematic categories:

1. Nutrition, addressing the nutritional composition of carob and its potential use as a cocoa substitute;
2. Pharmacological properties, focusing on reported antioxidant and anti-inflammatory activities based on experimental evidence;
3. Agricultural and environmental significance, examining the ecological role of the carob tree, its contribution to combating desertification, and its potential use in sustainable agroforestry systems.

This methodological approach enabled a structured and critical synthesis of current knowledge on *Ceratonia siliqua*, its traditional uses, and its potential applications in health, nutrition, and sustainable agriculture.

## 3. Overview

### 3.1. Botany

The carob tree (*Ceratonia siliqua* L., Fabaceae) is widely recognized as a characteristic species of the Mediterranean region. Mature trees can reach heights of up to 15 m, with a trunk circumference ranging from 2 to 3 m [19]. Young carob trees are characterized by smooth, grey bark that gradually becomes rough and brown with age [20]. As the tree matures, the wood color shifts from yellowish-white to dark red, making it suitable for furniture production and charcoal manufacturing [13].

**Figure 1:**
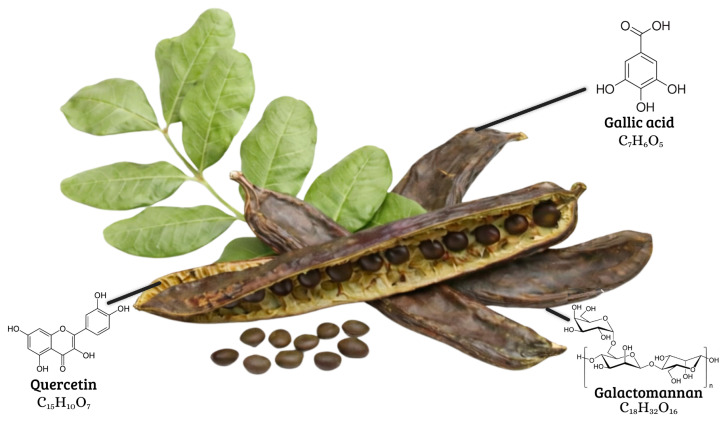
Bioactive compounds in different parts of the carob tree (*Ceratonia siliqua* L.) [2].

The leaves are leathery and glossy on the adaxial surface, while the abaxial surface is pale green. They typically measure 10 and 20 cm in length [21,22]. The flowers are unisexual, occurring as either male or female, and are arranged in complex inflorescences that display clear morphological differences between the two types [23]. The fruit is an indehiscent pod measuring between 10 and 30 cm in length; it is initially green but turns dark brown at maturity [24,25]. The seeds are oval, extremely hard, and historically served based on standardized units of weight, underscoring the long-standing economic importance of this species since antiquity [4] (Figure 1).

### 3.2. Origin of the Carob Tree (Ceratonia siliqua)

**Figure 2:**
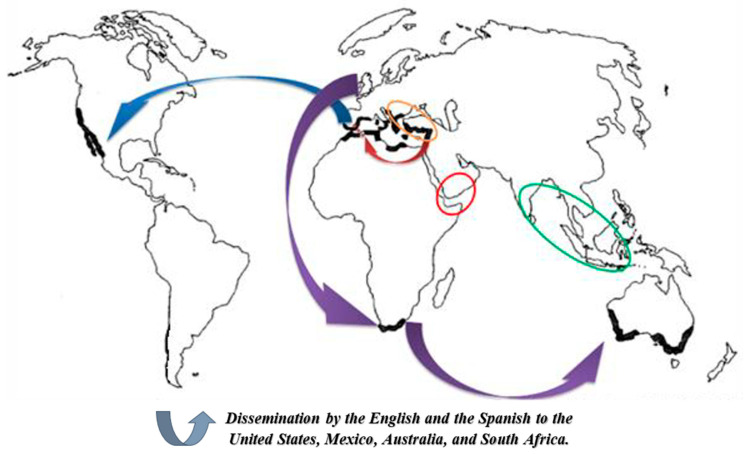
Center of origin and distribution of the carob tree across the world (the circles represent the different hypotheses that exist about the center of origin of *C. siliqua*, while the arrows symbolize the distribution of the species around the world) [2].

The geographical origin of the carob tree, as shown in Figure 2, has been debated, and several hypotheses have been proposed:

- Vavilov’s theory (1951) suggests that *Ceratonia siliqua* originated in the eastern Mediterranean region, including Turkey, Syria, and Palestine. This hypothesis is supported by historical and botanical evidence [26].
- Archaeobotanical evidence of wood analysis remains and charred fruits indicates the presence of carob trees in the mountainous regions of the southern Arabian Peninsula (Yemen) as early as 4000 BC [27,28]. The occurrence of this species on high plateaus, combined with its thermophilic characteristics, supports the hypothesis of an origin in this region.
- Zohary’s hypothesis (1999) proposes that the carob tree may represent a remnant of Indo-Malaysian flora, sharing common ancestors with genera such as Olea and Laurus [29,30]. This theory is supported by physiological traits, including late flowering (July–October) and C4 photosynthesis, which is typical of species adapted to warm climates. The exceptional durability of carob leaves further supports the possibility of a tropical origin.

### 3.3. Genetic Resources and Diversity in Carob (Ceratonia siliqua L.): Cultivars and Conservation Efforts in the Mediterranean Region

#### 3.3.1. Genetic Variability and Domestication

The carob tree has been cultivated for centuries using propagation techniques such as seeds, cuttings, and grafting [31]. Wild populations have historically provided the genetic foundation for cultivar selection and orchard development. Genetic studies indicate that cultivated carob exhibits relatively low divergence from its wild ancestors [32]. Nevertheless, modern cultivars display improved agronomic traits, including enhanced growth vigor, pod and seed quality, yield stability, and resistance to biotic stress [33,34].

#### 3.3.2. Major Cultivars and Selection Criteria

Carob cultivars are predominantly female, with only a limited number of male or hermaphroditic trees maintained for pollination. Traditional selection has emphasized fruit traits such as pod size, pulp thickness, sugar content, fiber yield, and seed-to-pod ratio, all of which are critical for industrial applications in the food and cosmetic sectors [35,36].

**Figure 3:**
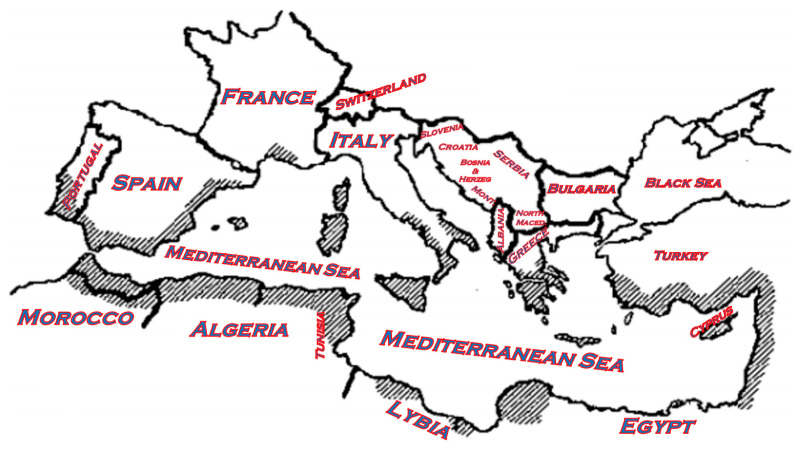
Distribution of carob tree cultivation areas in the Mediterranean basin [2,4].

Despite modest genetic differentiation from wild populations, carobs show considerable potential for cultivar improvement, particularly in Algeria, where breeding and reforestation initiatives are expanding. Strengthening germplasm conservation and selection programs could further enhance carob’s role in sustainable agriculture and rural economic development across Mediterranean regions [37] (Figure 3).

### 3.4. Global and Especially Economic Interests

*Ceratonia siliqua* holds considerable economic importance owing to the high commercial value of its fruits, especially the pods and seeds. Carob flour, derived from pod pulp, carob gum (E410), extracted from seeds, is widely used in the food industry. Carob gum is prized for its thickening, gelling, and stabilizing properties and is commonly incorporated into products such as sauces and ice cream. The global carob gum market was valued at approximately USD 103 million in 2020 and is projected to reach USD 121.9 million by 2026 [38]. Algeria ranks as the world’s fourth-largest producer, contributing about 5.99% to total output. This sector holds significant economic potential, particularly through the export of carob gum, which is experiencing growing demand in international markets. Beyond traditional uses, carob contributes substantially to agri-food economic development in Algeria and across the world [39,40].

### 3.5. Traditional Uses of Carob

The carob tree yields a wide range of products derived from its pods, pulp, and seeds, as summarized in Table 1 [41,42]. The pulp is commonly used as animal feed for horses and ruminants and is also processed for human consumption into sugar, alcohol, and dietary supplements [43,44].

Carob powder is produced through traditional processes involving washing, drying, roasting, and molding. It is widely used in culinary applications as a cocoa substitute and is incorporated into dietary and pharmaceutical formulations [3,45]. The seeds consist of three distinct components: the endosperm, embryo, and seed coat. Each of these has specific applications. The endosperm is processed into food additives, the embryo is processed into germ flour, and the seed coat is used for tannin extraction in leather processing [44,46].

**Table 1:** Uses and treatments of pulp and seed derivatives of the carob tree.

Products		Treatment Received	Uses
Pulp	Raw	None	Animal feed (horses and ruminants) [47]
	Molding	None	Human and animal feed (ruminants and non-ruminants) [48]
	Extraction and purification	None	Sugar and molasses [48]
	Fermentation and distillation	None	Alcohol and microbial protein production [49]
Powder	Washing, drying, roasting, molding	None	Food ingredients; cocoa substitute; preparation of dietary and pharmaceutical products [48]
Seeds	Endosperm	Molding	CBG or E-410; food additives; dietary fiber; pet food; pharmaceutical and cosmetic products [48]
	Embryo	Molding	Germ flour; human and animal nutrition [49]
	Seed coat	Extraction	Tannins for leather tanning [50,51]

### 3.6. Traditional Use in Medicine

Carob pods, leaves and bark have been recognized for their therapeutic properties, and they have been used for generations in traditional medicine to treat a wide range of conditions. Among the most frequently reported uses are anti-diarrheal effects attributed to tannin content (Table 2), which promote stool thickening and mucosal protection [52]. Experimental studies suggest that carob consumption may reduce total and LDL cholesterol levels, potentially benefiting cardiovascular health [53,54]. Additionally, carob extracts have demonstrated anti-inflammatory activity in experimental models [54]. Tannin-rich pods have historically been employed to relieve stomach discomfort and intestinal disorders, particularly diarrhea. Clinical and experimental studies suggest that carob may shorten the duration of diarrheal episodes, particularly in children [55,56].

Carob has also been traditionally consumed to alleviate abdominal pain, enteritis, and other gastrointestinal conditions, and is often prepared as herbal infusions [57,58]. Polyphenols and other bioactive compounds in carob extracts have demonstrated antioxidant and anti-inflammatory properties in experimental studies, suggesting potential protective effects against chronic diseases, such as cardiovascular disorders and certain cancers [59,60].

In addition to its medicinal value, carob is considered a nutritious food due to its high fiber content, which supports digestive health and may help regulate cholesterol and glucose levels. In many cultures, carob preparations are also used to soothe coughs and sore throats, owing to their demulcent properties. Overall, traditional medicinal uses of carob remain relevant today and are increasingly supported by scientific research, although further clinical validation is required [57,61,62].

**Table 2:** Chemical composition of bioactive components in *Ceratonia siliqua* L.

	TPC (mg EAG/g dw)	TFC (mg EQ/g dw)	TC (mg/g dw)	TAA (mg trolox/g dw)	A G µg/100 mg dw
Ethanol Extract of Carob Pods [63,64]	69.18 ± 0.15	34.88 ± 0.08	24.05 ± 0.15	41.62 ± 0.20	569.42
Leaves (Ethanolic Extract) [65]	163.50 ± 9.25	22.23 ± 8.61	/	DPPH IC_50_: 0.293 mg/mL Total Capacity: 189.01 µg AAE/mg	/
Leaves (Methanolic, Wild) [66]	6.19 ± 0.25	3.17 ± 0.64	/	ABTS: 0.46 mg TE/g DPPH: 0.29 mg TE/g	/
Leaves (Methanolic, Domesticated) [66]	4.23 ± 0.20	2.42 ± 0.36	/	ABTS: 0.37 mg TE/g DPPH: 0.19 mg TE/g	/
Pulp (Acetone Extract) [67]	53.22–183.31	1.41–7.46	/	/	/

TPC: Total Phenolic Content; TFC: Total Flavonoid Content; TAA: Total Antioxidant Activity; TC: Total Carotenoids; EQ: Quercetin Equivalent (QE); TE: Trolox Equivalent; dw: Dry Weight; Ascorbic Acid Equivalent (AAE); DPPH: 2;2-diphenyl-1-picrylhydrazyl; ABTS: 2;2′-azino-bis(3-ethylbenzothiazoline-6-sulfonic acid).

### 3.7. Bioactive Compounds and Medicinal Properties

Carob is well known for its rich content of bioactive compounds, which underpin its nutritional and medicinal value [62,63,64,65,66,67,68]. The concentration and composition of these compounds are influenced by factors such as cultivar type, processing methods, and extraction conditions. For example, variations in maceration and fermentation during traditional carob liquor production can significantly affect bioactive compound levels and final product quality [69,70,71,72].

#### 3.7.1. Active Substances of Biological Origin

Carob pods contain a multitude of bioactive compounds, such as the following:

- **Polyphenols:** Flavonoids and phenolic acids are among the most extensively studied constituents. These compounds exhibit antioxidant, anti-inflammatory, and antimicrobial activities in experimental models [68,73]. Their primary biological role is associated with protection against oxidative stress, which may reduce the risk of chronic diseases such as cardiovascular disorders and cancer [74,75]. Gallic acid and dietary fibers such as pectin are considered key contributors to the health-promoting effects of carob [76,77].
- **D-Pinitol:** A cyclitol derived from carob, D-pinitol has been reported to improve insulin sensitivity and support blood glucose regulation in experimental studies, suggesting its potential relevance for diabetes management [78,79,80,81,82].
- **Galactomannans:** There is an abundance of galactomannans in carob seeds; they are associated with cholesterol- and glucose-lowering effects and may contribute to reductions in LDL cholesterol and triglyceride levels [83,84]. Carob seeds also contain oleic and linoleic acids, which are known for their cardioprotective and anti-inflammatory properties [85,86].
- **Pectin fibers:** These fibers are present in carob pulp and play an important role in regulating intestinal transit. By forming a gel in the digestive tract, pectin can slow stool passage and is traditionally used to prevent diarrhea. Additionally, pectin contributes to satiety and supports gut microbiota health, potentially aiding weight management and digestive function [87,88].

#### 3.7.2. The Healing Virtues

- Antioxidant and anti-inflammatory potential

Gallic acid (48–56 mg/g dry matter), one of the major polyphenols identified in carob, exhibits strong antioxidant activity, exceeding that of vitamin E in vitro (ORAC = 12,500 μmol TE/g). Recent studies have shown that gallic acid can reduce interleukin-6 (IL-6) expression by approximately 40% and inhibit reactive oxygen species (ROS) production in Caco-2 cells by up to 75% [89,90]. In the context of identifying natural compounds capable of mitigating oxaliplatin-induced neuropathy, several studies have investigated immature carob pods and highlighted their phenolic composition and pronounced antioxidant activity [91,92]. Owing to these properties, carob-derived polyphenols may contribute to the prevention of diseases associated with oxidative stress, although further validation is required [81].

b. Glycemic control: validated mechanisms

D-pinitol, the predominant cyclitol present in carob pods, acts as an insulin-mimetic compound. A randomized clinical trial conducted in 2024 (*n* = 60) reported that supplementation with 500 mg/day of D-pinitol resulted in an 18% reduction in postprandial blood glucose levels in individuals with type 2 diabetes. This effect was attributed to the activation of the PI3K/Akt signaling pathway [93,94]. These findings suggest that D-pinitol may represent a promising natural adjuvant in antidiabetic therapeutic strategies.

c. Fibers: Dual Digestive and Cardiometabolic Action

Galactomannans extracted from the carob endosperm exhibit dose-dependent physiological effects. In terms of gut health, daily consumption of 15 g has been demonstrated to reduce intestinal transit time by approximately 40% and to accelerate recovery from pediatric infectious diarrhea [95,96]. Concerning lipid metabolism, three clinical trials reported an average reduction of 12% in LDL cholesterol levels [97,98]. In spite of this, the magnitude of LDL reduction varied widely, ranging from 7.4% to 22.5%.

These discrepancies may be attributed to differences in carob product composition, dosage, duration of intervention, participant health status (healthy individuals versus those with hypercholesterolemia or obesity), and variations in study design, including follow-up periods and run-in phases [97,98]. Overall, carob pulp supplementation is frequently associated with significant decreases in total and LDL cholesterol levels, although outcomes remain heterogeneous.

d. Fatty acids and cardiovascular prevention

Metabolic dysfunctions associated with increased cardiovascular risk require targeted nutritional strategies. Accumulating evidence from both preclinical and clinical studies highlights carob-derived products as promising agents with cardiometabolic benefits.

In a murine study, long-term supplementation (26 weeks) with a carob-based extract significantly attenuated metabolic alterations induced by an obesogenic diet, leading to a 17% reduction in blood glucose levels and marked improvements in lipid profiles, including a 13% decrease in total cholesterol and a 24% reduction in LDL cholesterol (*p* < 0.05) [99]. These changes were accompanied by a significant decrease in circulating IL-6 levels. At the vascular level, carob extract supplementation prevented diet-induced hypertension, restored endothelial function, reduced post-ischemic contractile dysfunction, and decreased cardiomyocyte apoptosis by more than 40% (*p* < 0.05) [99].

Complementarily, carob seed oil, characterized by a high oleic acid content (55–58%) and a balanced ω-6/ω-3 ratio of 2.3, demonstrated cardiovascular benefits comparable to olive oil. In a 2023 crossover clinical study, eight weeks of carob seed oil consumption resulted in a 21% reduction in C-reactive protein levels in patients with coronary disease [100,101].

### 3.8. Carob in Sustainable Forestry Systems

#### 3.8.1. Potential for Restoring Salt and Desert Regions

*Ceratonia siliqua* is among the Mediterranean species capable of surviving in arid and saline environments. Recent studies evaluating its germination capacity and growth performance under hydric and saline stress conditions support its suitability for the remediation of degraded soils [102]. Seedlings derived from seeds exposed to moderate salinity (≤200 mM NaCl) exhibited enhanced root robustness, which is a key trait for their establishment in acidic and degraded soils [103,104].

#### 3.8.2. Resistance to Salinity and Water Stress

Studies examining the combined effects of salinity and water scarcity on carob seed germination indicate that although high salt concentrations—particularly NaCl—can inhibit germination, seeds retain substantial germination potential under moderate stress conditions [104]. Notably, NaCl exerts lower inhibitory effects compared with other salts, suggesting a specific adaptation of carob to saline coastal soils prevalent in the Mediterranean region [105,106]. This tolerance highlights the relevance of carob for reforestation programs in arid areas with limited rainfall and restricted irrigation capacity.

### 3.9. Implications for Ecological Restoration

The carob tree presents considerable potential for ecological restoration in degraded arid environments [107]. Its ability to withstand prolonged drought and salinity stress makes it a valuable species for combating desertification and stabilizing saline soils [108]. This species’ adaptability provides a distinct advantage for restoring fragile ecosystems, particularly in regions where conventional tree species fail to be established. Incorporating carob into restoration initiatives supports soil conservation, enhances biodiversity, and contributes to sustainable agricultural landscapes in Mediterranean arid zones [105].

### 3.10. Interest in Animal Feed

Carob pulp and seeds have long been used in animal nutrition due to their high fiber, polyphenol, and natural sugar content (Table 3) [109,110]. Recent studies have evaluated their effects across multiple animal species [111,112]. Supplementation with carob pulp combined with vitamin E has demonstrated synergistic benefits in lambs, including enhanced rumination activity, improved antioxidant status, and favorable modulation of intestinal immune markers, without adverse effects on growth performance [113,114].

#### 3.10.1. Effects on Ruminants: Improvement of Rumination and Intestinal Health

Studies reported in [67,115] showed that diets enriched with carob pulp and vitamin E improved rumination behavior and strengthened antioxidant and immune defenses in lambs. Additionally, authors in [111,116] observed that incorporating carob into lamb diets reduced parasitic infections, including coccidiosis, and improved gut morphology, potentially decreasing the need for antibiotic treatments.

#### 3.10.2. Economic Profitability

Economic evaluations have indicated that partial substitution of corn with carob pulp in lamb diets yields modest growth effects but provides substantial benefits for digestive health, particularly within organic production systems [117]. Carob-based diets increased polyunsaturated fatty acid content while reducing lipid oxidation products, without negatively affecting oxidative stability or sensory quality [118,119].

#### 3.10.3. Human Food: A Functional Ingredient

The food industry has shown increasing interest in carob-derived ingredients, particularly carob seed germ flour, a protein-rich byproduct obtained during the extraction of locust bean gum (E410), as shown in Table 3. This ingredient exhibits notable nutritional value due to its high content of apigenin C-di- and poly-glycosylated derivatives, which have been associated with antioxidant and metabolic effects in experimental studies [120,121].

Experimental evidence suggests that the combination of carob-derived products and vitamin E can reduce reactive oxygen species (ROS) levels in the digestive tract and modulate inflammatory markers in the intestinal tissues of lambs, indicating a protective effect against oxidative stress induced by high-concentrate diets. Although these findings originate from animal models, they provide insight into the functional potential of carob-based ingredients.

Moreover, the presence of bioactive compounds such as tannins and flavonoids suggests that carob germ flour may exert antidiabetic effects. In vitro studies have reported inhibition of pancreatic α-amylase activity ranging from 40% to 60%, indicating a possible mechanism for modulating postprandial glucose levels [120,121,122,123]. Nevertheless, further clinical investigations are required to confirm these effects in human populations.

#### 3.10.4. Enriched Pasta with Reduced Glycemic Index

Pasta formulations enriched with carob germ flour, rich in C-glycosides, have been shown to inhibit carbohydrate-digesting enzymes, potentially contributing to improved glycemic regulation [122,123,124,125]. These properties make such products attractive for individuals who are overweight or have diabetes [118].

#### 3.10.5. Natural Sugar and Cocoa Substitute

Carob pulp, characterized by natural sweetness and low-fat content, is widely used as a cocoa substitute in bakery products, beverages, and desserts [126,127,128,129,130,131]. In contrast to cocoa, carob lacks caffeine and theobromine, making it suitable for children and stimulant-sensitive individuals [132,133]. Carob-based products are increasingly available to consumers and associated with health benefits [134,135]. However, significant variation in pod composition across regions highlights the need for long-term studies examining the influence of climate and soil conditions on phenolic and sugar content [136].

#### 3.10.6. Fibers and Antioxidants

Carob pulp is rich in soluble fibers, particularly carob gum, which enhances digestion and contributes to cholesterol reduction. Its polyphenol content further supports its role as a functional food ingredient [133,137]. Consumption of carob fiber has been associated with significant reductions in acylated ghrelin (49.1%), triglycerides (97.2%), and non-esterified fatty acids (67.2%), suggesting beneficial effects on fatty acid oxidation, energy intake, and body weight regulation [133].

## 4. Discussion

The nutritional and therapeutic potential of *Ceratonia siliqua* L. has been extensively investigated in recent years, yet reported outcomes show considerable variation across studies [1,2]. These inconsistencies reflect differences among cultivars, environmental conditions, genetic diversity, and phenotypic plasticity [1,2,26]. This discussion examines key contradictions regarding hypoglycemic effects, chemical composition variability, and pharmacological limitations, aiming to provide a coherent framework for interpreting existing data.

In vitro studies consistently demonstrate mechanisms underlying hypoglycemic activity, including inhibition of digestive enzymes, as discussed above, by aqueous carob extracts [5]. These findings align with the known effects of carob polysaccharides, particularly galactomannans, which increase intestinal viscosity, delay gastric emptying, and slow glucose absorption [9].

However, in vivo and clinical results remain inconsistent [43,63]. This discrepancy may be explained by the complex composition of carob pulp, in which polyphenols and fibers exert glucose-lowering effects, but high soluble sugar content may counteract these benefits [66]. Additionally, fiber type plays a critical role: insoluble fibers are more closely linked to lipid metabolism, whereas soluble viscous fibers primarily influence glycemic regulation [9,53,62].

Genetic and phenotypic studies, particularly on Algerian germplasm, reveal that *C. siliqua* comprises distinct populations with biochemical profiles strongly shaped by ecotype and abiotic stress [1,2,3,4]. Extraction parameters further contribute to variability by selectively influencing compound yields [8,68]. Future research should prioritize standardized documentation of cultivar, geographic origin, and extraction methods, alongside targeted clinical trials using chemically characterized extracts, to transform variability into evidence-based valorization [13,14,42,138].

## 5. Conclusions

*Ceratonia siliqua* is a multifunctional species of significant ecological, nutritional, and economic importance. Its high tolerance to drought and salinity makes it a valuable candidate for ecological restoration and desertification control in Mediterranean regions. In animal nutrition, carob-derived products have been shown to support digestive health and improve product quality, while potentially reducing dependence on synthetic additives. In human nutrition, the high fiber content and diversity of bioactive compounds present in carob offer promising perspectives for the development of functional foods aimed at supporting metabolic health and balanced diets.

The genetic diversity and long history of use of the carob tree further emphasize its cultural and economic relevance. Although an increasing number of experimental and applied studies support its therapeutic potential, further research—particularly well-designed and standardized clinical trials—is recommended to fully substantiate its health-related applications. Future development strategies should integrate genetic improvement, sustainable forestry practices, and circular economy approaches to enhance the valorization of carob while ensuring the conservation of Mediterranean ecosystems.

## Figures and Tables

**Table 3:** Commercial products derived from different morphological parts of the carob tree.

Product Category	Commercial Product	Part Used	Main Applications
Human Food [5,9]	Carob powder and carob syrup.	Milled fruit pulp.	Cocoa substitute (caffeine-free), natural sweetener, and high in fiber.
Dietary Supplements and Health [5,6]	Carob extract capsules and fiber supplements.	Pulp extract and purified fiber.	Digestive health with cholesterol-lowering effect (per fiber studies).
Food Industry [9]	Carob bean gum (E410) and thickener.	Seed endosperm (galactomannan).	Thickening, stabilizing, and gelling agent in ice cream, sauces, etc.
Animal Feed [9]	Carob pulp meal in ruminant feed.	Dried and milled pulp after seed removal.	Energy and fiber source in feed formulations.

## Data Availability

No new data were created or analyzed in this study. The data used in this work were obtained from publicly available sources, including scientific publications and ethnobotanical studies, which are cited in the manuscript. Therefore, data sharing is not applicable.

## Abbreviations

The following abbreviations are used in this manuscript:

LDL	Low-Density Lipoprotein
ROS	Reactive Oxygen Species
NaCl	Sodium chloride
CRP	C-Reactive Protein
mg/day	Milligrams Per Day
PI3K	Phosphoinositide 3-Kinase
CBG	Carboxylic Binding Gum
E-410	Locust bean gum
